# Long-term visual acuity in patients with optic pathway glioma treated during childhood with up-front BB-SFOP chemotherapy—Analysis of a French pediatric historical cohort

**DOI:** 10.1371/journal.pone.0212107

**Published:** 2019-03-08

**Authors:** Josué Rakotonjanahary, Nicolas Gravier, Julien Lambron, Emilie De Carli, Frédérique Toulgoat, Matthieu Delion, Isabelle Pellier, Xavier Rialland

**Affiliations:** 1 Department of Pediatric Oncology, University Hospital, Angers, France; 2 Kervision - Polyclinique de l’Atlantique, Nantes-Saint Herblain, France; 3 Department of Pediatric Radiology, Robert Debre Hospital, Assistance Publique-Hôpitaux de Paris (AP-HP), University Paris Diderot, Sorbonne Paris Cité, France; 4 Department of Radiology, University Hospital, Nantes, France; 5 Department of Neurosurgery, University Hospital, Angers, France; 6 UMR Inserm 892 - CNRS 6299 - CRCNA, Angers, France; University of Bern, SWITZERLAND

## Abstract

**Background:**

Visual outcome is one of the main issues in the treatment of optic pathway glioma in childhood. Although the prognostic factors of low vision have been discussed extensively, no reliable indicators for visual loss exist. Therefore, we aimed to define initial and evolving factors associated with long-term vision loss.

**Methods:**

We conducted a multicenter historical cohort study of children treated in France with up-front BB-SFOP chemotherapy between 1990 and 2004. Visual acuity performed at the long-term follow-up visit or within 6 months prior was analyzed. Logistic regression analysis was used to estimate the effects of clinical and radiological factors on long-term visual outcome.

**Findings:**

Of the 180 patients in the cohort, long-term visual acuity data were available for 132 (73.3%) patients (median follow-up: 14.2 years; range: 6.1–25.6). At the last follow-up, 61/132 patients (46.2%) had impaired vision, and 35 of these patients (57.3%) were partially sighted or blind. Multivariate analysis showed that factors associated with a worse prognosis for long-term visual acuity were an age at diagnosis of < 1 year (OR 3.5 [95% CI: 1.1–11.2], p = 0.04), tumor extent (OR 4.7 [95% CI: 1.2–19.9], p = 0.03), intracranial hypertension requiring one or more surgical procedures (OR 5.6 [95% CI: 1.8–18.4], p = 0.003), and the need for additional treatment after initial BB-SFOP chemotherapy (OR 3.5 [95% CI: 1.1–11.9], p = 0.04). NF1 status did not appear as a prognostic factor, but in non-NF1 patients, a decrease in tumor volume with contrast enhancement after BB-SFOP chemotherapy was directly associated with a better visual prognosis (OR 0.8 [95% CI: 0.8–0.9], p = 0.04).

**Interpretation:**

Our study confirms that a large proportion of children with optic pathway glioma have poor long-term outcomes of visual acuity. These data suggest new prognostic factors for visual acuity, but these results need to be confirmed further by large- and international-scale studies.

## Introduction

Optic pathway glioma (OPG) accounts for approximately 5% of brain tumors in children [1], and in approximately 30% of cases, OPG is associated with neurofibromatosis type 1 (NF1) [2,3]. A large majority of OPGs are pilocytic astrocytomas [3] and known to have a slow but unpredictable course. Although the 5-year survival rate is good, survival gradually decreases beginning at 10 years and never stabilizes [4]. Survivors experience multiple sequelae, especially neurological, visual and endocrine sequelae, which are likely to affect the quality of life in adulthood. Possible damage to the visual pathway and its impact on vision are the major issues of these tumors, and these possible outcomes are often a primary reason for initiating treatment. The tumor location does not allow for total resection except when the lesion is limited to a single optic nerve [5], and chemotherapy is often the first-line treatment applied in these young patients [6,7,8,9,10]. Although numerous reports have aimed to assess prognostic factors for visual outcomes, few have examined the very-long-term evolution (more than 10 years after diagnosis) of visual outcome, and the conclusions of these studies are often divergent. Indeed, they often differ significantly in terms of the study population (NF1 or non-NF1), treatments applied (or not applied), duration of follow-up, or criteria used to assess visual acuity (VA). Therefore, in this study, we focused on very-long-term VA in a homogenous population with the goal of answering two questions. What is the frequency of low vision in patients treated during childhood for OPG with up-front chemotherapy? Are there any initial or evolving factors (clinical or radiological) associated with very-long-term vision loss? We aimed to study this very-long-term visual impact in a historical cohort of patients treated with up-front BB-SFOP (Baby Brain-French Society of Pediatric Oncology) chemotherapy [10].

## Patients and methods

### Patients

This study is a historical cohort of patients who were less than 16 years of age when diagnosed with OPG and who were treated in France between June 1990 and December 2004 with BB-SFOP chemotherapy as the first-line treatment in the 33 French Society of Pediatric Oncology centres. This cohort has been previously described [4]. Patients were required to meet the two following criteria: i) be under 16 years old at diagnosis and ii) have been treated with up-front chemotherapy according to the BB-SFOP (Baby Brain SFOP) protocol. Partial surgery or shunting was possible before the initiation of chemotherapy. Children who initially underwent gross-total or sub-total resection as well as children who initially received radiotherapy or chemotherapy that was different from the BBSFOP regimen were excluded from the study.

Patients were invited to participate in a long-term follow-up visit. For each patient, we reviewed clinical data from diagnosis to the date of the last follow-up, including all treatments (chemotherapy, surgery, radiotherapy) applied during the evolution of disease. All parents or legal guardians of the patients signed informed consent forms before the assessments. The study was approved by an independent ethical research comitee (Comitee for the de Protection of Persons–CPP Ouest 3, Poitiers).

### Visual acuity outcomes assessment

We used a measure of the visual acuity dating at a maximum of 6 months before the long-term follow-up visit. Otherwise, the patients were reconvened for the assessment. The last values of VA were used for deceased patients or those who were lost to follow-up when available. VA was measured with the Snellen acuity chart for both eyes, and bilateral vision was categorized according to the World Health Organization (WHO) classification as WHO 0: mild or no visual impairment (≥ 3/10 or < 0.52 Log MAR); WHO 1: moderate visual impairment (< 3/10 and ≥ 1/10 or > 0.52 and ≤ 1 Log MAR); WHO 2: severe visual impairment (< 1/10 and ≥ 1/20 or > 1 Log MAR and ≤ 1.30 Log MAR); WHO 3: profound visual impairment (< 1/20 and ≥ 1/50 or > 1.30 and ≤ 1.69 Log MAR); WHO 4: near blindness (< 1/50 and light perception or > 1.69 and ≤ 1.9 Log MAR and light perception); or WHO 5: blindness (no light perception, 0/10 or 2.0 Log MAR). Patients were divided into 2 distinct groups according to the WHO classification: the first group corresponded to patients with WHO scores of 0, 1 or 2, and the second group corresponded to patients with WHO scores of 3, 4 or 5. This last group was designated as partially sighted or blind people.

### Clinical and radiological factors

The candidate risk factors for long-term visual acuity outcomes were described in our previous report [4]. They included clinical criteria at diagnosis (age < 1 year, sex, year of diagnosis [before or after 2000], and intracranial hypertension [ICHT] defined by clinical symptoms combined with radiological hydrocephalus, diencephalic syndrome [DS], defined by failure to thrive and a diagnostic weight between the 2nd and the 5th percentiles, and NF1 status) and the use of subsequent treatment after BB-SFOP chemotherapy (surgery and/or radiotherapy and/or other chemotherapy regimen).

We also collected magnetic resonance images (MRI) at diagnosis, at the end of BB-SFOP chemotherapy and at the last follow-up. The MRI scans were reviewed by two radiologists using the previously described classification [11] with quantitative and qualitative evaluations as follows: lesions were scored as 0 (no lesion), 1 (unilateral lesion) or 2 (bilateral lesion). A chiasmatic lesion, which, by definition, cannot be bilateral, must be noted 1 [11]. The sum of each of these scores led to a global extension score between 1 (single chiasma lesion) and 17 (Table 1) [11]. As we previously explained [11], the MRI review was limited to non-NF1 patients to avoid possible errors related to the existence of non-neoplastic lesions in NF1 patients. For non-NF1 patients, radiological analysis included MRI outcomes at diagnosis and at the end of BB-SFOP chemotherapy, including qualitative and quantitative data and the extents of the initial lesions. For non-NF1 patients, when possible, we calculated the total tumour volume and the tumour volume with contrast enhancement at diagnosis and at the end of BB-SFOP [11]. The extent of the initial lesion was assessed using qualitative MRI data as described above. The median value of the extent of the initial lesion (corresponding to the number of lesions) was used as the reference. Finally, the long-term visual acuity outcomes were also adjusted for evolutive factors, such as the length of follow-up and vital status (living or deceased patients).

**Table 1:** Qualitative analysis of MRI.

optic nerve ^(1)^	0	1	2	
prechiasmatic ^(2)^	0	1	2	
chiasmatic	0	1		
retrochiasmatic ^(3)^	0	1	2	
optic tracts ^(4)^	0	1	2	
hypothalamic involvement ^(5)^	0	1		
pituitary gland involvement ^(6)^	0	1		
pituitary stalk involvement ^(7)^	0	1		
brainstem involvement ^(8)^	0	1		
forebrain involvement	0	1		
leptomeningeal dissemination ^(9)^	0	1		
ventricular dilatation ^(10)^	0	1		NA
other site of brain disease	0	1		NA

0: absence of involvement / 1: unilateral involvement / 2: bilateral involvement / NA: not applicable.

^(1)^ The optic nerve is limited by the orbital apex. The term refers to the first (intraocular) and second (intraorbital) segments.

^(2)^ Prechiasmatic refers to the third (intracanalicular) and fourth (intracranial) segments.

^(3)^ The anterior segment of the optic tracts is in front of the anterior portion of the cerebral peduncles.

^(4)^ The posterior segment of the optic tracts is behind the anterior part of the cerebral peduncle.

^(5)^ Hypothalamic involvement is evaluated on sagittal planes between the pituitary stalk and the mamillary bodies.

^(6)^ The pituitary gland is considered involved when it is thickened, when its limits cannot be observed, or when the sella turcica is involved.

^(7)^ The pituitary stalk is considered involved when it is not observed, when it is thickened or when it is included in the lesion.

^(8)^ Including the cerebral peduncles.

^(9)^ Meningeal enhancement apart from a postoperative context.

^(10)^ When there is no dilatation but a shunt is present, ventricular dilatation is considered not applicable (NA).

### Statistical analysis

Descriptive statistics were used to summarize the characteristics of the patients with OPG. Data included in the analysis were monitored from the time of diagnosis to March 30, 2016, or the date of the last follow-up, last contact or death. The median follow-up time was calculated for living patients.

Univariate and multivariate logistic regression analyses were used to calculate the odds ratios (ORs) and to estimate the effects of the potential risk factors on the risk for long-term blindness or partial sight (WHO 3, 4 and 5). For the logistic regression multivariate analysis, a step-down variable selection using Akaike’s information criterion was used as a stopping rule.

Some outcomes were not available for the whole cohort. Missing data can lead to a loss in statistical power and to potentially biased results if not handled appropriately [12]. Therefore, to evaluate the possibility that the restriction of the study population to participants with complete data induced bias [13,14], we repeated the analyses on the whole cohort and on non-NF1 patients [14]. Analyses on the whole cohort were performed using the MICE procedure (Multivariate Imputation by Chained Equations) [15] for the management of missing data. We assumed data were missing at random. The MICE procedure was used in Stata software for the management of missing data for long-term VA and MRI outcomes (quantitative and qualitative evaluation data).

A large number of imputed datasets (70) was created because a large number is preferable to reduce sampling variability from the imputation process [14]. As quantitative MRI outcomes were available only for non-NF1 patients [11], non-NF1 patients were analyzed separately using the MICE procedure.

All statistical tests were performed with a two-sided level of significance of 0.05. The statistical analysis was performed using Stata software 12.1.

## Results

**Fig 1:**
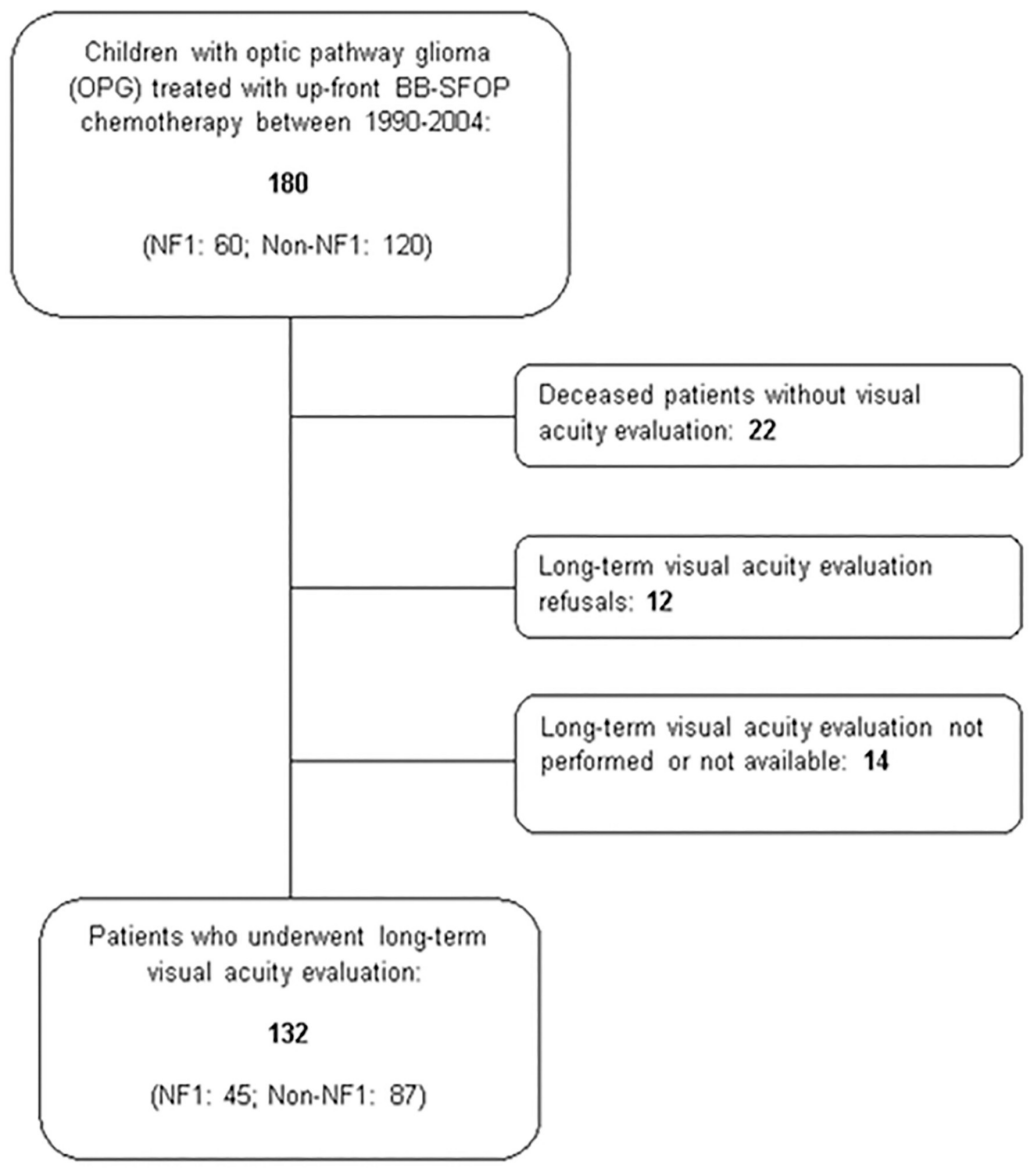
Children with OPG treated with up-front BB-SFOP chemotherapy: Flow diagram of the long-term visual acuity evaluation.

A total of 445 children received BB-SFOP chemotherapy in France between June 1990 and December 2004. Indications were various, and only 182 patients less than 16 years of age were observed as having been treated with BBSFOP chemotherapy as first-line treatment for OPG. Two patients could not be included in the analysis since too much diagnosis data were missing from their records [4]. Ultimately, our series included 180 children (120 non-NF1 patients (66.7%) and 60 NF1 patients (33.3%), with a median follow-up of 14.3 years (range: 6.1–25.6). Of the 180 patients in the cohort, 132 (73.3%) could be analyzed for long-term VA: 22 were dead without VA assessment, and 26 refused a VA assessment or were lost to follow-up (Fig 1). At the last follow-up, 61/132 patients (46.2%) had impaired vision (WHO ≠ 0), and among these patients, 35/61 (57.3%) were partially sighted or blind, corresponding to a visual handicap according to French legislation. Unilateral blindness was noted in 25/132 patients (18.9%): eighteen patients rated 0 in the WHO classification, six patients rated 1 or 2, and one patient rated 3.

The results were then analyzed in two different ways corresponding to the two statistical methodologies described above: the first corresponded to the statistical analysis of the 132 patients with VA, and the second corresponded to the statistical analysis of the 180 patients of the cohort and of the 120 non-NF1 patients, including the patients with missing data in terms of VA.

### Results of the 132 patients who underwent long-term VA evaluations

Of these 132 patients, 86 (65.2%) had a sporadic OPG, and 46 (34.8%) had an NF1-associated OPG. The median follow-up time of this population was 14.2 years (range: 6.1–25.6). First, we analyzed the clinical criteria at diagnosis (Table 2). According to the univariate logistic regression analysis, age was the only factor correlated with a worse prognosis.

**Table 2:** Characteristics of patients at diagnosis.

Characteristics	Patients who underwent long-term VA evaluations^3^	All patients	Non-NF1 patients
	n = 132	n = 180	n = 120
**Sex**			
- male	62 (46.9%)	84 (46.6%)	54 (55%)
- female	70 (53.1%)	96 (53.4%)	66 (45%)
- sex ratio (M/F)	0.88	0.87	0.81
**Age** (years)			
- < 1	31 (23.5%)	45 (25%)	42 (35%)
- 1–4	76 (57.6%)	102 (56.7%)	56 (46.7%)
- 5–9	19 (14.4%)	24 (13.3%)	15 (12.5%)
- ≥ 10	6 (4.5%)	9 (5%)	7 (5.8%)
- Median (IQR)	2.6 (1–4.3)	2.4 (1–4.2)	1.6 (0.7–4)
**Year of diagnosis**			
- 1990–2000	70 (53.1%)	99 (55%)	65 (54.2%)
- > 2000	62 (46.9%)	81 (45%)	55 (45.8%)
**NF1**			
- yes	45 (34%)	60 (33.3%)	--
- no	87 (66%)	120 (66.7%)	
**Diencephalic syndrome**^1^			
- yes	23 (17.4%)	32 (17.8%)	28 (23.3%)
- no	109 (82.6%)	148 (82.2%)	92 (76.7%)
**Intracranial Hypertension**^2^			
- yes	31 (23.5%)	48 (26.7%)	39 (32.5%)
- no	101 (76.5%)	132 (73.3%)	81 (67.5%)

^1^ Diencephalic syndrome was defined as failure to thrive and a diagnostic weight between the 2^nd^ and the 5^th^ percentiles.

^2^ Intracranial hypertension was defined by clinical symptoms associated to radiological hydrocephalus.

^3^ VA: Visual Acuity.

Among the 31 patients who were less than 1 year of age at diagnosis, 16 (51.7%) had WHO scores of 3, 4 or 5, whereas 19 (18.8%) who were over 1 year of age had these scores (p = 0.001). No significant differences were found for the other factors analyzed, especially the NF1 status.

Then, we analyzed various evolutive factors as previously described using univariate logistic regression analysis (Table 3). Deceased patients had a worse visual prognosis than living patients: 8/14 dead patients (57.1%) were partially sighted or blind versus 27/112 living patients (24.1%) (p = 0.01). A significant difference in long-term VA (WHO 0, 1 and 2 versus WHO 3, 4 and 5) was also found when patients received subsequent chemotherapy after BB-SFOP (p = 0.02), when partial resection was applied after BB-SFOP (p = 0.002), when tumor surgery was associated with radiotherapy (p = 0.02) or when any treatment (chemotherapy and/or radiotherapy and/or chemotherapy) was applied after the BB-SFOP regimen (p = 0.008). Finally, patients who underwent at least one surgical procedure for ICHT had a worse prognosis in terms of long-term VA (WHO 3, 4 or 5): 23/47 patients (48.9%) when one or more surgical procedures were necessary versus 12/85 patients (14.2%) when no surgery was necessary (p<10^−3^).

**Table 3:** Long-term visual acuity according to clinical and evolutive characteristics (univariate analysis).

Characteristics	All patients	Patients with VA	WHO	WHO	WHO	p	OR*	[95% CI]
	n = 180 (%)^a^	n = 132 (%)^a^	0, 1 and 2	3, 4 and 5	md			
			n = 97 (%)^b^	n = 35 (%)^b^	n = 48 (%)^c^			
**Sex**								
- male	84 (46.6%)	62 (46.9%)	48 (76.2%)	15 (23.8%)	21 (25%)	0.57	0.8	0.3–17
- female	96 (53.4%)	70 (53.1%)	49 (71%)	20 (29%)	27 (28.1%)			
- sex ratio (M/F)	0.87	0.88	0.98	0.75	0.85			
**Age** (years)						0.001	4.6**	1.9–10.9
- < 1	45 (25%)	31 (23.5%)	15 (48.3%)	16 (51.7%)	14 (31.1%)			
-5	102 (56.7%)	76 (57.6%)	62 (81.6%)	14 (18.4%)	26 (25.5%)			
- ≥ 5	33 (18.3%)	25 (18.9%)	20 (80%)	5 (20%)	8 (24.2%)			
- Median (IQR)	2.4 (1–4.2)							
**Year of diagnosis**								
- 1990–2000	99 (55%)	70 (53.1%)	49 (68.9%)	22 (31.1%)	28 (28.3%)	0.17	0.6	0.2–1.2
- ≥ 2000	81 (45%)	62 (46.9%)	48 (78.6%)	13 (21.4%)	20 (24.7%)			
**NF1**						0.1	0.4	0.2–12
- yes	60 (33.3%)	46 (34.8%)	38 (81.7%)	8 (18.3%)	14 (23.3%)			
- no	120 (66.7%)	86 (65.2%)	59 (68.6%)	27 (31.4%)	34 (28.3%)			
**Diencephalic syndrome**								
- yes	32 (17.8%)	23 (17.4%)	14 (60.9%)	9 (39.1%)	9 (28.1%)	0.13	2.0	0.8–5.2
- no	148 (82.2%)	109 (82.6%)	83 (76.1%)	26 (23.9%)	39 (26.3%)			
**Intracranial hypertension**								
- yes	48 (26.7%)	31 (23.5%)	23 (74.2%)	8 (25.8%)	17 (35.4%)	0.92	0.9	0.3–2.4
- no	132 (73.3%)	101 (76.5%)	74 (73.2%)	27 (26.8%)	31 (23.5%)			
**Length of follow-up (years)**								
- < 5	12 (6.7%)	2 (1.5%)	2 (100%)	0 (0%)	10 (83.3%)	0.17	1.0	0.9–12
- ≥ 5 et < 10	33 (18.3%)	20 (15.1%)	18 (90%)	2 (10%)	13 (39.4%)			
- ≥ 10 et < 15	88 (48.9%)	70 (53%)	48 (68.5%)	22 (31.5%)	18 (20.4%)			
- ≥ 15	47 (26.1%)	40 (30.4%)	29 (72.5%)	11 (27.5%)	7 (14.9%)			
**Evolution**								
- living	123 (68%)	118 (89%)	91 (77.1%)	27 (22.9%)	5 (4%)			
- deceased	36 (20%)	14 (11%)	6 (42.9%)	8 (57.1%)	22 (61%)	0.01	4.5	1.4–14.1
- lost to follow-up or refusals	21 (12%)	-		-	21 (100%)			
**Subsequent chemotherapy**								
0	83 (46.1%)	60 (45.5%)	50 (83.4%)	10 (16.6%)	23 (27.7%)	0.02	2.5	1.1–5.8
- 1 to 3	81 (45%)	59 (44.7%)	41 (69.5%)	18 (30.5%)	22 (27.2%)			
- 4 to 5	12 (6.7%)	10 (7.6%)	4 (40%)	6 (60%)	2 (16.7%)			
- ≥ 6	4 (2.2%)	3 (2.2%)	2 (66.7%)	1 (33.3%)	1 (25%)			
**Specific treatment other than chemotherapy**								
- Surgery (partial resection)	30 (16.7%)	22 (16.7%)	13 (59.1%)	9 (40.9%)	8 (26.7%)	0.002	1.5	1.6–8.1
- Radiotherapy	28 (15.6%)	20 (15.1%)	15 (75%)	5 (25%)	8 (28.6%)	0.07	2.1	0.09–4.6
- Surgery + Radiotherapy	27 (15%)	24 (18.2%)	13 (54.2%)	11 (45.8%)	3 (6.2%)	0.02	2.9	1.2–7.4
- No specific treatment other than chemotherapy	95 (52.7%)	66 (50%)	56 (84.8%)	10 (15.2%)	29 (30.5%)	0.004	3.4	1.5–7.8
**Surgical procedures for intracranial hypertension**								
- 0 surgical procedure	111 (61.7%)	85 (64.4%)	73 (85.8%)	12 (14.2%)	26 (23.4%)	<10^−3^	5.8	2.5–13.4
- 1 surgical procedure	20 (11.1%)	11 (8.3%)	5 (45.5%)	6 (54.5%)	9 (45%)			
- 2 to 4 surgical procedures	38 (21.1%)	29 (21.9%)	17 (58.6%)	12 (41.4%)	9 (23.7%)			
- ≥ 5 surgical procedures	11 (6.1%)	7 (5.4%)	2 (28.6%)	5 (71.4%)	4 (36.4%)			
**Treatment after BB-SFOP chemotherapy (second-line chemotherapy and/or surgery and/or radiotherapy)**								
- No treatment	55 (30.5%)	40 (30.3%)	37 (92.5%)	3 (7.5%)	15 (27.3%)	0.008	4.5	1.5–14
- Treatment	125 (69.5%)	92 (69.7%)	60 (65.2%)	32 (34.8%)	33 (26.4%)			

^a^: column percentages;

^b^: row percentages (n = patients with VA);

^c^: row percentages (n = all patients)

*WHO 0, 1 and 2 vs WHO 3, 4 and 5;

**< 1 vs ≥ 1

The results of the MRI analysis for non-NF1 patients (87/132) are summarized in Table 4. According to the univariate logistic regression analysis of MRI at diagnosis and after BB-SFOP, no association was found between radiological factors (the tumor volume or the location of the tumor) and long-term VA. When considering the extension score of the initial tumor (as previously defined), we found a median score of 7 (range: 2–13) and the extent of the initial lesion was the only factor observed as statistically significant. Patients with scores < 7 had better visual prognoses than those with scores ≥ 7. In each group, we respectively found 24/27 (88.9%) and 20/34 (58.8%) patients with WHO scores of 0, 1 and 2 versus 3/27 (11.1%) and 14/34 (41.2%) patients with WHO scores of 3, 4 or 5 (blindness or partial sight; p = 0.008, OR = 5.5 [95% CI: 1.6–19.3]).

**Table 4:** Long-term visual acuity in non-NF1 patients according to the initial size and location of the tumor.

Characteristics	All non-NF1 patients	Patients with VA	WHO 0, 1 and 2	WHO 3, 4 and 5	WHO md
	n = 120 (%)^a^	n = 86 (%)^a^	n = 59 (%)^b^	n = 27 (%)^b^	n = 34 (%)^c^
**Volume of total lesion**					
• < 30 000	18 (15%)	17 (19.8%)	14 (82.3%)	3 (17.7%)	1(5.6%)
• 30 000 − 60 000	19 (15.8%)	14 (16.3%)	12 (85.8%)	2 (14.2%)	5 (26.3%)
• ≥ 60 000	19 (15.8%)	15 (17.4%)	12 (80.0%)	3 (10%)	4 (21%)
• md	64 (53.4%)-	40 (46.5%)	21 (52.5%)	19 (47.5%)	24 (37.5%)
**Volume of solid portion with contrast enhancement**					
• < 30 000	21 (17.5%)	18 (20.9%)	16 (88.9%)	2 (11.1%)	3 (14.3%)
• 30 000–60 000	12 (10%)	9 (10.5%)	8 (88.9%)	1 (11.1%)	3 (8.8%)
• ≥ 60 000	10 (8.3%)	9 (10.5%)	7 (77.7%)	2 (22.3%)	1 (10%)
• md	77 (64.2%)	50 (58.1%)	28 (56%)	22 (44%)	27 (35.1%)
**Optic nerves**					
• 0	69 (57.5%)	56 (65.1%)	43 (76.7%)	13 (23.3%)	13 (18.8%)
• 1	4 (3.3%)	1 (1.1%)	0 (0%)	1 (100%)	3 (75%)
• 2	4 (3.3%)	4 (4.6%)	1 (25%)	3 (75%)	0 (0%)
• md	43 (35.9%)	25 (29.2%)	15 (60%)	10 (40%)	18 (41.9%)
**Prechiasmatic**					
• 0	9 (7.5%)	8 (9.3%)	7 (87.5%)	1 (12.5%)	1 (11.1%)
• 1	20 (16.7%)	14 (16.3%)	13 (92.8%)	1 (7.2%)	6 (30%)
• 2	48 (40%)	39 (45.3%)	24 (61.5%)	15 (38.5%)	9 (18.7%)
• md	43 (35.8%)	25 (29.1%)	15 (60%)	10 (40%)	18 (41.8%)
**Chiasmatic**					
• 1	77 (64.2%)	61 (70.9%)	44 (72.1%)	17 (27.9%)	16 (20.8%)
• md	43 (35.8%)	25 (29.1%)	15 (60%)	10 (40%)	18 (41.9%)
**Retrochiasmatic**					
• 0	3 (2.5%)	2 (2.3%)	2 (100%)	0 (0%)	1 (33.3%)
• 1	73 (60.8%)	58 (67.4%)	41 (70.6%)	17 (29.4%)	15 (20.5%)
• 2	1 (0.8%)	1 (1.2%)	1 (100%)	0 (0%)	0 (0%)
• md	43 (35.9%)	25 (29.1%)	15 (60%)	10 (40%)	18 (41.9%)
**Optic tracts**					
• 0	34 (28.3%)	26 (30.2%)	22 (84.6%)	4 (15.4%)	8 (23.5%)
• 1	42 (35%)	34 (39.5%)	21 (61.8%)	13 (38.2%)	8 (19%)
• 2	1 (0.8%)	1 (1.2%)	1 (100%)	0 (0%)	0 (0%)
• md	43 (35.9%)	25 (29.1%)	15 (60%)	10 (40%)	18 (41.9%)
**Ventricular dilatation**					
• 0	41 (34.2%)	34 (39.6%)	24 (70.5%)	10 (29.5%)	7 (17.1%)
• 1	35 (29.2%)	26 (30.2%)	19 (73.1%)	7 (26.9%)	9 (25.7%)
• md	44 (36.6%)	26 (30.2%)	16 (61.6%)	10 (38.4%)	18 (40.9%)
**Hypothalamic involvement**					
• 0	2 (1.6%)	2 (2.3%)	2 (100%)	0 (0%)	0 (0%)
• 1	75 (62.5%)	59 (68.7%)	42 (71.1%)	17 (28.9%)	16 (21.3%)
• md	43 (35.9%)	25 (29%)	15 (60%)	10 (40%)	18 (41.9%)
**Pituitary stalk involvement**					
• 0	53 (44.2%)	44 (51.2%)	34 (77.3%)	10 (22.7%)	9 (17%)
• 1	24 (19.9%)	17 (19.8%)	10 (58.8%)	7 (41.2%)	7 (29.2%)
• md	43 (35.9%)	25 (29%)	15 (60%)	10 (40%)	18 (41.9%)
**Pituitary gland involvement**					
• 0	75 (62.5%)	59 (71%)	44 (74.5%)	15 (25.5%)	16 (21.3%)
• 1	2 (1.6%)	0 (0%)	0 (0%)	2 (100%)	0 (0%)
• md	43 (35.9%)	25 (29%)	15 (60%)	10 (40%)	18 (41.9%)
**Brainstem involvement**					
• 0	67 (55.8%)	51 (59.4%)	38 (74.5%)	13 (25.5%)	16 (23.9%)
• 1	10 (8.3%)	10 (11.6%)	6 (60%)	4 (40%)	0 (0%)
• md	43 (35.9%)	25 (29%)	15 (60%)	10 (40%)	18 (41.9%)
**Forebrain involvement**					
• 0	33 (27.5%)	26 (30.2%)	22 (84.6%)	4 (15.4%)	7 (21.2%)
• 1	44 (36.6%)	35 (40.8%)	22 (62.9%)	13 (37.1%)	9 (20.4%)
• md	43 (35.9%)	25 (29%)	15 (60%)	10 (40%)	18 (41.9%)
**Leptomeningeal dissemination**					
• 0	74 (62%)	59 (68.6%)	43 (72.8%)	16 (27.2%)	15 (20.3%)
• 1	3 (2.1%)	2 (2.4%	1 (50%)	1 (50%)	1 (33.3%)
• md	43 (35.9%)	25 (29%)	15 (60%)	10 (40%)	18 (41.9%)
**Other sites of brain disease**					
• 0	75 (62.5%)	59 (68.6%)	43 (72.8%)	16 (27.2%)	16 (21.3%)
• 1	2 (1.6%)	2 (2.4%)	1 (50%)	1 (50%)	0 (0%)
• md	43 (35.9%)	25 (29%)	15 (60%)	10 (40%)	18 (41.9%)
**Number of tumoral locations**					
• < 7	32 (26.6%)	27 (31.4%)	24 (88.9%)	3 (11.1%)	5 (15.6%)
• ≥ 7	44 (36.7%)	34 (39.5%)	20 (58.8%)	14 (41.2%)	10 (22.7%)
• md	44 (36.7%)	25 (29.1%)	15 (60%)	10 (40%)	19 (43.2%)

md = missing data. /

^a^: column percentages;

^b^: row percentages (n = patients with VA);

^c^: row percentages (n = all patients).

Finally, when considering all factors analyzed and the results of the multivariate logistic regression analysis, only age (<1 year), the use of any specific treatment after BB-SFOP chemotherapy (other chemotherapy regimen and/or surgery and/or radiotherapy), the need for surgery for ICHT and number of tumor locations ≥ 7 were significantly associated with a worse prognosis for long-term VA (Table 5).

**Table 5:** Factors associated with long-term partial sight or blindness (WHO 3, 4 or 5): Multivariate analysis (patients who underwent long-term VA evaluations, N = 132).

	OR	p	[95%CI]
Age < 1 year	3.5	0.04	1.1–11.2
Surgery for ICHT	5.6	0.003	1.8–18.4
Number of tumoral locations at diagnosis ≥ 7	4.7	0.03	1.2–19.9
Treatment after BB-SFOP	3.5	0.04	1.1–11.9

### Results for the 180 patients (whole cohort)

We performed a second statistical analysis of the entire cohort of 180 patients according to the method described above to account for patients with missing data and to avoid reducing the analysis to a smaller subsample. According to the univariate logistic regression analysis, age < 1 year, specific treatments after BB-SFOP chemotherapy and ICHT requiring one or more surgical procedures were clinical and evolutive factors associated with a poor long-term outcome of VA. When considering the extension score of the tumor (median: 7 [range: 2–13]), a score ≥ 7 was also significantly associated with long-term blindness or partial sight (WHO 3, 4 or 5). According to the multivariate logistic regression analysis (Table 6), we found that only age < 1 year at diagnosis, the need for surgery for ICHT, and ≥ 7 tumor locations were significantly associated with a poor prognosis of long-term VA (WHO 3, 4 or 5).

**Table 6:** Factors associated with long-term partial sight or blindness (WHO 3, 4 or 5): Multivariate analysis (whole cohort, N = 180).

	OR	p	[95%CI]
Age < 1 year	2.3	0.05	1.1–5.7
Surgery for ICHT	2.8	0.01	1.2–6.7
Number of tumoral locations at diagnosis ≥ 7	4.4	0.02	1.2–16.5

### Results for the 120 non-NF1 patients

The median follow-up of this population was 14.4 years (range: 6.1–25.6). For all non-NF1 patients (120 patients), we applied the same methodology, taking into account missing data. According to the univariate logistic regression, age < 1 year at diagnosis, surgery for ICHT and an MRI score ≥ 7 were significantly associated with long-term blindness or partial sight (WHO 3, 4 or 5). According to the multivariate logistic regression analysis, age < 1 year at diagnosis was no longer associated with this poor prognosis. However, a response to BB-SFOP chemotherapy (evaluated based on a decrease in tumor volume with contrast enhancement regardless of the percentage of the decrease) appeared to be directly linked to better visual prognosis. Finally, the need for surgery for ICHT was the third factor correlated with a worse visual prognosis (WHO 3, 4 or 5). The results are summarized in Table 7.

**Table 7:** Factors associated with long-term partial sight or blindness (WHO 3, 4 or 5): Multivariate analysis (non-NF1 patients, N = 120).

	OR	p	[95%CI]
Surgery for ICHT	3.8	0.03	1.1–13.5
Number of tumoral locations at diagnosis ≥ 7	7.7	0.02	1.2–48.3
Decrease in tumor volume with contrast enhancement at the end of BB-SFOP	0.8	0.04	0.8–0.9

## Discussion

Here, we report the very-long-term visual outcomes of a cohort of 180 patients treated during childhood for OPG with up-front BB-SFOP chemotherapy. This is one of the largest series of such patients, and our study confirms that visual prognosis is one of the key issues for long-term survivors of OPG who are treated during childhood. More than 10 years after diagnosis, almost half of children have impaired VA (WHO ≠ 0), and among them, more than half are partially sighted or blind. Therefore, considering the legal classification applied in France, approximately 25% of patients treated for OPG during childhood have visual disability in adulthood.

Although this study is a historical study with all of the implied limitations, we could identify several clinical, radiological and evolutive factors associated with poor visual outcomes.

Various clinical factors at diagnosis have been mentioned as likely poor prognostic factors for long-term VA, among them age at diagnosis. Thus, in a cohort of 88 patients with NF1-associated OPG, Fisher et al. [16] found a trend for poor visual prognosis for patients less than 2 years of age and for those older than 5 years of age. In a cohort of 43 NF1 and non-NF1 patients, Dodgshun et al. [17] could not draw a conclusion but found that patients diagnosed in infancy were overrepresented in the legally blind group. Based on a cohort of 32 patients, Campagna et al. [18] suggested that children with a young age at diagnosis of NF1-associated OPG had an unfavorable visual outcome; however, the authors could not statistically test this hypothesis. In our study, we identified age < 1 year as a poor prognostic factor by analyzing the cohort of children who had an evaluation of VA and the entire cohort of 180 children, including patients with missing data. By contrast, in the cohort of non-NF1 patients, this factor appeared in univariate analysis but disappeared in multivariate analysis.

NF1 status is another factor mentioned as likely to affect long-term visual prognosis. From the results of a series of 59 patients with sporadic OPG and from a comparison of these results with those of other series evaluating the long-term VA of NF1-associated OPGs [18,19,20,21], Wan et al.[22], in 2016, suggested a better visual prognosis for NF1 patients. Nevertheless, we believe that such historical comparisons between series that differ in many criteria (number of patients, age, treatment applied, VA evaluation) are extremely difficult and do not allow reliable conclusions. By contrast, the patients we present here are a homogeneously treated and evaluated population, and we determined that there was no statistically significant difference in long-term VA between patients with sporadic OPG and NF1-associated OPG.

The relationship between imaging and, more specifically, tumor location as evaluated by MRI at diagnosis is the third prognostic factor studied by numerous authors. For a series of 43 patients with NF1-associated OPGs, Balcer et al.[19] showed that the involvement of optic tracts and post-chiasmatic structures was associated with a higher probability of vision loss. Similar conclusions were made by Wan et al.[22] who, in a cohort of 59 patients with sporadic OPGs, noted a worse visual prognosis in patients with post-chiasmatic involvement at diagnosis. Conversely, from a series of 44 patients with NF1-associated OPGs, Segal et al. [21] found no difference between retrochiasmatic gliomas and those limited to the optic nerve and chiasma. For their part, Dodghund et al. [17], in a series of 43 patients with NF1 and non-NF1 OPGs, found a statistically significantly higher risk of vision loss in patients with chiasmatic/hypothalamic tumors than in patients with tumors at other locations. After analyzing a cohort of 45 patients with NF1 and non-NF1 OPGs, Tow et al. [23] found that patients with involvement of both the optic chiasma and hypothalamus had the highest visual morbidity. Fisher et al. [16] found that the involvement of the optic tracts/radiations was the only significant prognostic factor for worsening of VA. These results seem contradictory and conflict with our own. In our large series, with very-long-term evaluation, no tumor location appeared as a prognostic factor for long-term VA, and this was true regardless of the population analyzed. However, the extent of the tumor appeared statistically correlated with a poor long-term visual prognosis when the extension score was greater than or equal to 7, although we did not find this correlation in the literature.

Two other results from our study are notable. First, ICHT requiring surgery was associated with a poor visual outcome in all multivariate analyses. This result may appear paradoxical as initial ICHT is not a factor for poor prognosis. We have no explanation for this difference, but one can imagine that the level of intracranial pressure and the compression of optic pathways (requiring one or more surgeries) that occur during ICHT contribute to the worsening of visual prognosis. Second, during our VA assessments of the 132 patients, we found that any treatment after BB-SFOP chemotherapy was statistically significantly associated with a poor long-term prognosis of VA. We presume that this result is comparable to the fact that the absence of a radiological response to treatment with BB-SFOP among non-NF1 patients is also a poor prognostic factor. Although our study does not demonstrate this finding, we can hypothesize that these two clinical and radiological parameters reflect the response to chemotherapy and that a lack of response is a predictor of poor outcome for VA.

This study has several limitations. First, we could not compare long-term VA with VA at diagnosis because, in the majority of cases, the latter was not accurately reported in the clinical data, probably due to the age of children at diagnosis (81.7% of the 180 patients were under 5 years of age at diagnosis) making it difficult, if not impossible, to accurately assess VA at this time (Table 2). Second, we required that all patients had an optical assessment minimally comprising funduscopic, visual field, color vision, ocular alignment and VA evaluations. Unfortunately, some ophthalmologists in the different centers did not perform this evaluation. While this assessment is very time-consuming, our study demonstrates that the assessment is absolutely necessary. For this reason, we limited the study to that of VA instead of extending it to the entire visual function. Third, we could not review all of the MRIs of the non-NF1 patients because many of the scans, at diagnosis or at the end of the BB-SFOP, were performed a long time ago (more than fifteen years) and were not available for reliable quantitative or qualitative tumor evaluations. This limitation highlights the difficulty in the management of these patients, the absolute necessity of cooperation between pediatric oncologists, neuro-oncologists, neuro-radiologists and ophthalmologists, and the need for the standardization of follow-up for these patients, not only at diagnosis but also throughout treatment and long after the end of treatment.

Additionally, in our analysis, multiple imputation procedures were used for the management of missing data. A large fraction of the data was imputed and observed, and imputed values were compared. We assumed that the data were missing at random, and although a complete case analysis of patients who had undergone long-term evaluation may suffer more chance variation, multiple imputation should correct biases that might arise in complete case analyses [14]. However, some differences between the results (such whether age <1 year is a prognostic factor) are difficult to explain. We consider that taking into account all of the variables and potential risk factors that were included in our analysis may increase the plausibility of the missing at random assumption. Even so, the missing data at random assumption should be considered carefully in such a long-term evaluation study because some data may not be missing at random. A sensitive analysis exploring the best and worse scenarios of long-term outcomes would have been useful if only a few values were missing [14].

In summary, despite its limitations, this study confirms that a large proportion of children with OPGs have poor long-term outcomes of VA. The data suggest that an age less than 1 year at diagnosis, the extent of lesions on MRI, ICHT requiring one or more surgeries, and a poor response to initial chemotherapy may be risk factors for vision loss. Further large-and international-scale studies are necessary to confirm these results, but the definition of specific risk factors today is a key point in the management of these patients, until new therapeutic approaches are developed that can improve vision loss [24,25].

## Supporting information

S1 Dataset Long-term visual acuity according to the WHO classification. (ZIP)
