# Supplementary material for: Long-term visual acuity in patients with optic pathway glioma treated during childhood with up-front BB-SFOP chemotherapy—Analysis of a French pediatric historical cohort
Source: PLoS One. 2019 Mar 8;14(3):e0212107. doi: 10.1371/journal.pone.0212107 (PMC6407847; doi:10.1371/journal.pone.0212107)
Supplement: S1 Dataset — (ZIP) [file pone.0212107.s001.zip › S1 Dataset_Read me.pdf]

Supporting information File:

S1 Dataset. Long-Term Visual Acuity According to the WHO classification.

- ID (column 1) : Patient ID
- VA_WHO (column 2) : Visual acuity according to the World Health Organization (WHO) Classification
- WHO_345 (column 3) : Partially sighted or blind patients (WHO 3, 4 and 5)
- WHO_012 (column 4) : WHO 0, 1 and 2
- WHO_impaired (column 5) : Patients with impaired vision (WHO ≠ 0)
- U_Blind (column 6) : Unilateral Blindness
